# Factors associated with unmet fertility desire and perceptions of ideal family size among women in Bangladesh: Insights from a nationwide Demographic and Health Survey

**DOI:** 10.1371/journal.pone.0233634

**Published:** 2020-05-22

**Authors:** Raisul Akram, Abdur Razzaque Sarker, Nurnabi Sheikh, Nausad Ali, MGN Mozumder, Marufa Sultana

**Affiliations:** 1 Bangladesh Institute of Development Studies (BIDS), Agargaon, Dhaka, Bangladesh; 2 Department of Management Science, University of Strathclyde, Glasgow, United Kingdom; 3 Nutrition and Clinical Services Division, International Centre for Diarrheal Disease Research, Dhaka, Bangladesh; 4 Deakin Health Economics, School of Health and Social Development, Deakin University, Geelong, Victoria, Australia; Anglia Ruskin University, UNITED KINGDOM

## Abstract

**Introduction:**

Along with the developing world, Bangladesh has made a tremendous improvement in declining total fertility rate, however, this declining trend is not uniform to all the socio-demographic stratum. Incongruities exist between the numbers of children that women bearing and what they actually desired which refers to unmet fertility desire. This study aims to elicit women’s perception of ideal number of children and predictors of unmet fertility desire in Bangladesh.

**Method:**

This study analyzed nationally representative cross-sectional Bangladesh Demographic and Health Survey 2014 data. A two-stage stratified random sampling technique was used while a total of 17,863 ever-married women were interviewed between June and November 2014. A total of 10,912 eligible women were included in the analysis. Poisson regression analysis and logistic regression models were used to measure women’s perception of the ideal number of children and to determine the influencing factors of unmet fertility desires.

**Result:**

The mean value of the perceived ideal number of children was 2.22 (SD ± 0.73) and the majority of women (71.2%) expect to have two children in their lifetime. Approximately 46% of mothers reported bearing more children than they desired. The perceived ideal number of children was significantly higher among women who were living in rural areas, from Sylhet division, Muslim, unemployed, and experienced child death and those who justified beating. Findings revealed that several factors such as place of residence, geographic location, religion, wealth index, maternal age and education, partners’ education, experiencing child death, and other empowerment-related indicators were significantly associated with unmet fertility desires.

**Conclusion:**

Perceived ideal number of children differs among women’s socioeconomic and demographic strata. Unmet fertility desire was also found which indicates that reproductive knowledge and health care services are still necessary for some socio-demographically disadvantaged/vulnerable people and this group should be regularly monitored to control population growth.

## Introduction

There is ample evidence of declining total fertility rate (TFR) worldwide including developing countries. For instance, TFR had declined globally from 4.98 to 2.4 from 1960 to 2017, almost half by 60 years [1]. In line with the other developing countries, Bangladesh has also experienced a decreasing trend of TFR over decades with a current TFR of 2.3 in 2014 [2]. This noticeable decline in TFR is often attributed to a successful family planning program including the use of contraceptive initiatives targeting the women [3]. Despite the extensive coverage of family planning interventions including the use of contraceptives, fertility desires among women and TFR vary widely across different geographic and socio-economic strata in Bangladesh [2,4,5]. Fertility desires and childbearing intentions are the most significant approaches that determine fertility behavior which is an important predictor of future population growth of a country. On the other hand, the unmet need for fertility or unmet fertility desire refers to the situation when the actual number of bearing children is greater or less than the number of children she desired to have. In that cases when the actual number of children is more than the desired, it has an impact on the TFR. Therefore, TFR is induced by the unmet fertility desire while it is believed that elimination of unmet fertility desire would substantially decline TFR. Although a body of literature focused on the unmet need for fertility and its associated factors, such studies are limited in Bangladeshi context [6–10]. In addition, family planning 2030 agenda of sustainable development goals (SDGs) has also focused on the challenges of unmet fertility desires despite substantial achievement that has already been made in expanding access to contraception. To ensure universal access to reproductive health services, particularly for developing countries like Bangladesh, together with family planning (goal 3), gender equality and women empowerment (goal 5) were also included in order to achieve the agenda by 2030 [11].

Many studies have identified the association of women’s socio-economic and decision-making autonomy with the use of contraceptives, intimate partner violence, and health-care services on fertility decline. However, none of them focused on fertility desires and determinants of unmet fertility needs in the Bangladeshi context. For instance, Duvendack and colleagues (2016) showed the association of women empowerment and the trend of fertility among women by years’ span [5]. AMF Rabbi (2014) sorted out the fertility preference and concluded that the expected time interval for the next children plays a crucial role in the TFR scenario in Bangladesh [12]. Some other studies generally focused on the relationships of women's decision making autonomy with either the use of contraception or maternal health care services [6,7,10,13–22].

Based on the available literature, it is evident that assessing the determinants of fertility intension and exploring the extent to which they are associated with having more than desired children are crucial for the performance of family planning programs initiatives and for the population policy of a country [12]. Many studies around the world have conceptualized different pathways that affect fertility preference and identified socio-economic and demographic factors of fertility preferences and actual demand for children. For instance, using a conceptual framework, Upadhyay and colleagues identified how women’s fertility is affected by their socio-demographic and empowerment-related indicators in the Sub-Saharan Africa context [18]. A study from Nigeria figured out that timing of childbearing, level of education, economic status, place of residents as well as experience of child death were significantly associated with unmet fertility desires [23]. In addition, many research findings have shown that women’s involvement in household decisions making has an impact on family planning services and childbearing decisions [6–8,13–15]. Although women’s autonomy is a multidimensional concept, in short, it conveys a set of discrete components or phenomena essential for ensuring that women can exercise their rights with full potential [6].

Despite the growing importance of fertility desires and unmet need, little is known about their actual desires, unmet fertility need with associated predictors including women's autonomy related indicators are rarely found in the context of Bangladesh using nationwide survey data. To mitigate these gaps, this study intended to get further insights into the current fertility desires with unmet fertility needs in terms of women’s perception of the ideal number of children by disaggregating socio-demographic, and women's autonomy related indicators. Analysis from the latest nationwide demographic and health survey data would thus allow us for generating evidence on how the fertility preference is distributed across socio-demographic groups and by women's autonomy related factors. It is expected that the evidence generated from the findings would be useful for family planning policy implication targeting the most appropriate population of Bangladesh.

## Materials and methods

### Data source

Bangladesh Demographic and Health Survey (BDHS) is a part of the long-standing worldwide Demographic and Health Survey (DHS) program which is conducted every 3 years and captures information covering individual and household-level health and demographic data nationwide. This study used the latest (seventh) round of nationally representative BDHS 2014 dataset for analysis. A wide range of information was collected through questionnaire-based, face-to-face interviews, where reproductive-age women (15–49 years) were interviewed based on the MEASURE DHS program model [24]. A two-stage stratified random sampling technique was used in this survey. This survey used the sampling frame provided by the Bangladesh Bureau of Statistics, which was previously used for the Population and Housing Census 2011 conducted in Bangladesh. This survey was conducted between June and November 2014, by a trained and experienced data collection team. A total of 18,000 residential households were selected for the survey, and a total of 17,863 interviews were completed among respective women with a 98% response rate [2]. All DHS data are publicly accessible and were made available upon request by MEASURE DHS. Furthermore, approval was sought from and given by the MEASURE DHS program office to use this dataset.

### Explanatory variables

Explanatory variables were selected based on a literature review, prior knowledge, and the availability of variables in the Bangladesh Health and Demography Survey (BDHS) 2014 dataset. These variables included the area of residence, administrative divisions, wealth quintile, religious view, respondent age, respondent education, partner education, currently living children, experience with child death, types of contraception use, involvement in household decision making, beating justification (the attitude of women toward being beaten by their husband), NGO membership, control over earnings, and access to mass media. In this analysis, categorization was performed for several continuous variables. Since it is well established that maternal age is an important factor in reproduction, and as of many other earlier studies maternal age was categorized by five years of interval. This will allow getting insights into the change of fertility behavior of women of different age groups. Therefore, respondent’s age was categorized into five groups (15–25 years, 26–30 years, 31–35 years, 36–40 years, and over 40 years). The education level of the women and their partners were categorized into four groups: “no education,” “primary”, “secondary”, and “higher”. No education refers to not attaining any formal education, while primary education is defined as completing grade 5, secondary as completing grade 10, and higher as attaining more than a grade 10 education. We utilized the predetermined wealth index category provided with the dataset, which was generated from selected household assets using principal component analysis (PCA) and classified into five groups: “poorest”, “poorer”, “middle”, “rich”, and “richest”. The types of contraception use were categorized into three groups: “no method”, “traditional method” and “modern method”. No method is defined as neither the respondent nor her husband using any contraception during intercourse, while the traditional method comprises of periodic abstinence, and the modern method includes the birth control pill, injectable birth control, condoms, male or female sterilization, intrauterine contraceptive device (IUD), and implants.

The women’s empowerment-related variables used in this analysis include the number of household decisions in which women participated, attitude toward being beaten by their husband, membership in any NGOs, and control over income. To access women’s decision-making ability, they were asked five questions: participation in decision making regarding i) their own health care, ii) major household purchases, iii) child’s health care, iv) visits to their family and relatives, and v) using contraception. Attitude toward family violence (e.g., being beaten by their husband), which was presented as beating justification and categorized as either “yes” or “no”. Beating justification was categorized as yes if the woman justifies beating by her husband for any of five reasons (i.e., if goes outside without telling her husband, neglects her children, argues with her husband, refuses to have sex, or burning food). If the woman justifies beating for none of the following reasons, then it was categorized as no. Moreover, membership in any NGOs and involvement in any income generation activities with control over her earnings were also categorized and included in the analysis.

### Outcome variables

The outcome variables of the study include “perceived ideal number of children” and “unmet fertility desire”. For this study, we analyzed data of those women who wanted no more children, were sterilized or declared infecund, and provided numerical answers for fertility desire (ideal number of children). These criteria were used to restrict the sample to women who have theoretically completed their reproductive age meaning that according to their statement they are unable or strongly unwilling to conceive as of their current status. These restrictions yielded a sample size of 10,912 eligible participants. The DHS dataset gathered information on the women’s perceptions of the ideal number of children by asking different questions to the respondent(s). Women with living children were asked, “If you could go back to the time when you did not have any children and could choose exactly the number of children to have in life, how many would that be?” Women with no children at the time of the survey were asked, “If you could choose exactly the number of children to have in life, how many would that be?” Another outcome variable, “unmet fertility desire”, that means having more children than they desired was defined as the difference between the desired and actual number of children a respondent had during the time of the survey [23]. The variable was generated in accordance with earlier relevant studies and was calculated by subtracting the number of living children from the desired or perceived ideal number of children of the same respondent [10,18,23]. This computation provided two types of unmet fertility desire: having more children than desired and having fewer children than desired. For this current study “unmet fertility desire” was considered for those who had exceeded their desired number of children and was coded as “1” and “0” otherwise.

### Statistical analysis

Datasets were checked for missing values and outliers prior to analysis. A proper sampling weight provided with the dataset by MEASURE DHS was applied in this analysis to make the sample more representative of the population across different areas of the country. Descriptive statistics, such as frequency distribution in terms of percentages and 95% confidence interval (CI) were estimated to outline the background characteristics of study participants. The association between women’s perception of the ideal number of children and selected demographic and empowerment-related variables was investigated using the Poisson regression model. Two logistic regression models; Model I and Model II were constructed to predict the association of socio-demographic variables with women’s unmet fertility desires. Model I stands for the bivariate logistic regression model representing the crude association of dependent variable with each of the independent variables (unmet fertility desire) where the Model II constructed the multivariate logistic regression model while all of the explanatory variables were adjusted simultaneously with the dependent variable. Variables that showed a significant association in the bivariate logistic regression analysis were added into the multivariate logistic regression model. Diagnostic tests were employed in the analysis. Variance Inflation Factor (VIF) was calculated to detect multicollinearity in the model. The low value of average VIF for both the Poisson regression (3.09) and logistic regression (2.98) confirms no notable multicollinearity among variables. In the Poisson regression analysis, the goodness-of-fit chi-squared test is not statistically significant that indicated that the model fitted reasonability well. For the logistic regression model, linear predicted value (_hat) and linear predicted value squared (_hatsq), determined using *linktest* in Stata 14.0 confirm that the constructed model was well specified where the large p-value of the logistic regression model (*p* = 0.352) obtained from the Hosmer-Lemeshow test statistics of goodness-of-fit indicates the acceptance of the model. Data cleaning, validation, and all statistical analyses were performed using Stata/SE 14.0 (Stata Corporation, College Station, TX, USA).

### Ethical approval

BDHS 2014 is a publicly available dataset and can be downloaded from the DHS Program website (https://dhsprogram.com/data/available-datasets.cfm). We analyzed the dataset after receiving approval from the MEASURE DHS program office. The survey followed standardized data collection procedures and received ethics approval from the National Research Ethics Committee (NREC) of the Bangladesh Ministry of Health and Family Welfare. According to the DHS, written informed consent was obtained from all participants before they enrolled in the survey.

## Results

### Background characteristics of study participants

The description and distribution of study participant characteristics for this analysis are presented in Table 1. Among the women (n = 10,912), approximately two-thirds (73%) of them lived in urban areas, with approximately 33.8% of them originating from the Dhaka division, followed by Chittagong (17.4%). Notably, the majority of participants were Muslim (89.1%). The highest percentage (22.4%) of participants were 30–34 years of age, followed by 25–29 years (18.6%), and 35–39 years (18.1%). The participation of respondents from all five economic groups was approximately equal (around 20% for each). Only 5.8% of the women had more than secondary level education, while 31% had no formal education. Approximately one-third of the partners (34%) of study participants had no formal education and 12.5% of their partners had higher than the secondary level of education. Over half of the participants (58.2%) reported using modern contraception methods (either themselves or their partner), while 31.7% reported using no method to prevent pregnancy. Twenty-one percent of respondents reported that they previously had experience with child death. More than half (59.1%) of the respondents had access to mass media. Substantial variations were observed for the women’s empowerment-related indicators among participants. The findings revealed that less than one-third (31.9%) of women had participated in all the five household decision-making activities, while 6.2% of women did not participate in any of the household decisions, and 40.5% reported their involvement in making three to four decisions out of five. The results further indicated that 71.4% of women did not justify beating for any of the indicated reasons. Furthermore, 38.3% of respondents had a membership with NGOs and one-third (34.3%) were involved in income-generating activities, among which 4.7% had no control over their earnings. The mean value of the perceived ideal number of children was 2.22 (SD ± 0.73), where the expectation of having two children was the highest (71.2%) and 46.2% of mothers reported bearing more children than they desired.

**Table 1:** Percentage distribution of study participants by demographic and empowerment characteristics (n = 10,912).

Characteristics of Sample	Percentage (%) (n = 10,912)	95% CI	
		*Lower*	*Higher*
***Socio-demographic***			
**Area of residence**			
*Urban*	27.04	26.22	27.89
*Rural*	72.96	72.11	73.78
**Division**			
*Rajshahi*	12.63	12.02	13.27
*Barisal*	6.26	5.82	6.73
*Chittagong*	17.36	16.66	18.08
*Dhaka*	33.75	32.87	34.64
*Khulna*	11.13	10.56	11.74
*Rangpur*	12.41	11.80	13.04
*Sylhet*	6.46	6.01	6.94
**Wealth quintile**			
*poorest*	19.95	19.21	20.71
*poorer*	20.13	19.38	20.89
*middle*	19.91	19.17	20.67
*richer*	20.09	19.35	20.85
*richest*	19.93	19.19	20.69
**Religion**			
*Muslim*	89.06	88.46	89.63
*Hinduism*	9.09	8.56	9.64
*Others*	1.86	1.62	2.13
**Respondent age (in years)**			
*15–19*	1.87	1.63	2.14
*20–24*	9.90	9.36	10.48
*25–29*	18.61	17.89	19.35
*30–34*	22.41	21.64	23.21
*35–39*	18.06	17.34	18.79
*40–44*	16.10	15.42	16.80
*45–49*	13.05	12.44	13.70
**Respondent education**			
*No formal education*	30.98	30.12	31.86
*Primary*	31.60	30.73	32.48
*Secondary*	31.62	30.75	32.5
*Higher*	5.80	5.38	6.25
**Partner education**			
*No formal education*	33.99	33.11	34.89
*Primary*	27.31	26.49	28.16
*Secondary*	26.20	25.38	27.03
*Higher*	12.49	11.89	13.13
**Currently living children**			
*2 or less*	45.36	44.43	46.3
*3–4 children*	43.35	42.43	44.29
*More than 4 children*	11.28	10.7	11.89
**Ever experienced child death**			
*No*	79.1	78.32	79.85
*Yes*	20.9	20.15	21.68
**Types of contraception use**			
*No method*	31.66	30.79	32.54
*Traditional*	10.13	9.58	10.71
*Modern*	58.21	57.28	59.13
**Access to mass media**			
*No*	40.87	39.95	41.79
*Yes*	59.13	58.21	60.05
***Women empowerment***			
**Number of decision(s) women participate**			
*0*	6.18	5.75	6.65
*1–2*	21.38	20.62	22.16
*3–4*	40.49	39.58	41.42
*5*	31.94	31.08	32.83
**Beating Justification**			
*No*	71.35	70.49	72.19
*Yes*	28.65	27.81	29.51
**Member of any NGO**			
*No*	61.69	60.77	62.59
*Yes*	38.31	37.41	39.23
**Respondent's control over earnings**			
*No earning*	65.68	64.78	66.56
*No control*	4.66	4.28	5.07
*Control*	29.66	28.81	30.53
***Fertility desire***			
**Unmet fertility desire**			
*More than desired children*	46.21	45.27	47.14
*Less or equal than desired children*	53.79	52.86	54.73
**Perception on ideal number of children**			
*Not more than 1 child*	7.27	6.8	7.78
*2 children*	71.21	70.36	72.05
*more than 2 children*	21.51	20.75	22.29
**Mean value for ideal number of children** *(mean ± SD)*		***2*.*22 (± 0*.*73)***	

CI: confidence interval; NGO: non-government organization; SD: standard deviation; No formal education, primary, secondary, and higher education refers to not attaining any formal education, completing grade 5, grade 10, and completing higher than grade 10, respectively.

### Associated factors for the ideal number of children

Results from the linear regression model describe the association of women’s perceptions of the ideal number of children with selected demographic and empowerment-related variables (Table 2). The findings revealed that several factors, such as place of residence and geographic location, religion, wealth index, age, education, experiencing child death, and certain empowerment indicators (i.e., beating justification, control over earnings, and access to mass media) were significantly associated with mothers’ desired number of children. Overall, rural mothers and those from the Sylhet division expect a higher number of children compared to urban mothers and those from the Rajshahi division (coefficient: 0.18, CI: 0.15, 0.21; *p-value*: *<0*.*001*). The results revealed that Hindu mothers desired fewer children compared to Muslims (coefficient: -0.08, CI: -0.1, -0.06; *p-value*: *<0*.*001*). Increasing coefficient values were also observed among mothers with increasing age; notably, we observed the highest coefficient values for mothers aged 45 to 49 years and the lowest for the 20–24 age group (coefficients: 0.45 and 0.21, respectively) compared to the mother aged 15–19 years, which implies that elderly mothers desired more children than younger mothers. Additionally, mothers from the poorest households and those with lower educational attainment expected a higher number of children than the richest and those with higher educational attainment. Also, mothers who experienced child death desired more children than their counterparts (coefficient: 0.04, CI: 0.02, 0.06; *p-value <0*.*001*).

**Table 2:** Coefficients from the poisson regression analysis examining the perceived ideal number of children by socio-demographic and empowerment characteristics.

Characteristics of Sample	Coefficient (95% CI)	Robust SE	*p-*value
***Socio-demographic***			
**Area of residence**			
*Urban®*	-		
*Rural*	0.04 (0.02, 0.06)	0.01	**<0.001**
**Division**			
*Rajshahi®*	-		
*Barisal*	0.08 (0.05, 0.10)	0.01	**<0.001**
*Chittagong*	0.15 (0.13, 0.18)	0.01	**<0.001**
*Dhaka*	0.05 (0.03, 0.07)	0.01	**<0.001**
*Khulna*	-0.02 (-0.04, 0.01)	0.01	0.141
*Rangpur*	0.02 (0.01, 0.05)	0.01	**0.045**
*Sylhet*	0.18 (0.15, 0.21)	0.02	**<0.001**
**Wealth quintile**			
*poorest*	0.05 (0.02, 0.08)	0.02	**0.003**
*poorer*	0.03 (0.01, 0.06)	0.01	**0.025**
*middle*	0.02 (0.00, 0.05)	0.01	0.077
*richer*	0.04 (0.01, 0.06)	0.01	**0.003**
*richest®*	-		
**Religion**			
*Muslin®*	-		
*Hinduism*	-0.08 (-0.1, -0.06)	0.01	**<0.001**
*Others*	-0.1 (-0.17, -0.02)	0.04	**0.009**
**Respondent age (in years)**			
*15–19 ®*			
*20–24*	0.21 (0.13, 0.30)	0.40	**<0.001**
*25–29*	0.28 (0.20, 0.37)	0.40	**<0.001**
*30–34*	0.35 (0.27, 0.43)	0.40	**<0.001**
*35–39*	0.39 (0.30,0.47)	0.40	**<0.001**
*40–44*	0.40 (0.31, 0.49)	0.40	**<0.001**
*45–49*	0.45 (0.36, 0.53)	0.40	**<0.001**
**Respondent education**			
*No formal education*	0.10 (0.06, 0.14)	0.02	**<0.001**
*Primary*	0.07 (0.03, 0.10)	0.02	**0.001**
*Secondary*	0.04 (0.01, 0.08)	0.02	**0.022**
*Higher®*	-		
**Partner education**			
*No formal education*	0.01 (-0.04, 0.03)	0.02	0.825
*Primary*	0.01 (-0.03, 0.03)	0.02	0.902
*Secondary*	-0.02 (-0.04, 0.01)	0.01	0.273
*Higher®*	-		
**Ever experienced child death**			
*No®*	-		
*Yes*	0.04 (0.02, 0.06)	0.01	**<0.001**
**Types of contraception use**			
*No method*	0.01 (-0.01, 0.03)	0.01	0.255
*Traditional*	0.01 (-0.02, 0.04)	0.01	0.397
*Modern®*	-		
**Access to mass media**			
*No*	0.03 (0.01, 0.05)	0.01	**0.012**
*Yes®*	-		
***Women empowerment***			
**Number of decision(s) women participate**			
*0*	0.04 (-0.01, 0.08)	0.02	0.110
*1–2*	0.01 (-0.02, 0.03)	0.01	0.644
*3–4*	0.01 (-0.02, 0.02)	0.01	0.733
*5®*	-		
**Beating Justification**			
*No®*	-		
*Yes*	0.02 (0.01, 0.04)	0.01	**0.008**
**Member of any NGO**			
*No*	0.01 (0.00, 0.03)	0.01	0.104
*Yes®*	-		
**Respondent's control over earnings**			
*No earning*	0.03 (0.01, 0.05)	0.01	**0.002**
*No control*	0.02 (-0.01, 0.06)	0.02	0.196
*Control®*	-		

*®*: Reference category; CI: confidence interval; SE: standard error

Regarding the empowerment indicators, a desire for more children was observed among mothers who justified beating for any reason (coefficient: 0.02, CI: 0.01, 0.04; *p-value = 0*.*008*), those who were not involved in any income-generating activities (coefficient: 0.03, CI: 0.01, 0.05; *p-value = 0*.*002*), and those who do not have the access to any mass media (coefficient: 0.03, CI: 0.01, 0.05; *p-value = 0*.*012*).

### Factors associated with unmet fertility desire

Findings from the logistic regression analysis presented in Table 3 shows both the unadjusted and adjusted effects of socio-demographic and empowerment-related variables with the unmet fertility desire of respondents. Based on the unadjusted model (Model I), the highest odds ratio of unmet needs (more children than desired) was observed among rural women, those from the Sylhet division, and individuals in lower economic groups. Furthermore, the highest odds ratio was found among elderly women, those with lower educational attainment (both respondent and partner), and among Muslim respondents. Mothers who experienced child death and used no contraceptive methods were also found to have more children than they desired. Regarding the empowerment indicators, mothers who participated in three to four household decisions were members of an NGO, and those who had access to mass media were more likely to have more than their desired number of children.

**Table 3:** Logistic regression analysis of associated factors for unmet fertility desires among women by socio-demographic and empowerment characteristics.

Characteristics of Sample	Unadjusted model (Model I)^1^		Adjusted model (Model II)^2^	
	OR (95% CI)	*p-*value	OR (95% CI)	*p-*value
***Socio-demographic***				
**Area of residence**				
*Urban ®*	1.00		1.00	
*Rural*	1.39 (1.27, 1.51)	**<0.001**	1.11 (0.99, 1.23)	0.067
**Division**				
*Rajshahi ®*	1.00		1.00	
*Barisal*	1.51 (1.26, 1.82)	**<0.001**	1.59 (1.30, 1.95)	**<0.001**
*Chittagong*	1.69 (1.47, 1.94)	**<0.001**	2.32 (1.98, 2.73)	**<0.001**
*Dhaka*	1.29 (1.14, 1.46)	**<0.001**	1.47 (1.28, 1.69)	**<0.001**
*Khulna*	1.07 (0.91, 1.25)	0.420	1.12 (0.95, 1.33)	0.181
*Rangpur*	1.13 (0.97, 1.31)	0.119	1.13 (0.96, 1.34)	0.140
*Sylhet*	2.02 (1.68, 2.43)	**<0.001**	2.20 (1.80, 2.70)	**<0.001**
**Wealth quintile**				
*Poorest*	1.95 (1.73, 2.21)	**<0.001**	1.60 (1.32, 1.92)	**<0.001**
*Poorer*	1.64 (1.46, 1.86)	**<0.001**	1.19 (1.00, 1.41)	0.056
*Middle*	1.45 (1.28, 1.63)	**<0.001**	1.09 (0.93, 1.28)	0.274
*Richer*	1.26 (1.12, 1.42)	**<0.001**	1.01 (0.87, 1.16)	0.936
*richest ®*	1.00		1.00	
**Religion**				
*Muslim ®*	1.00		1.00	
*Hinduism*	0.6 (0.53, 0.69)	**<0.001**	0.58 (0.5, 0.67)	**<0.001**
*Others*	0.5 (0.37, 0.68)	**<0.001**	0.31 (0.22, 0.43)	**<0.001**
**Respondent age (in years)**				
*15–19®*	0.02 (0.01, 0.05)	**<0.001**	0.02 (0.01, 0.04)	**<0.001**
*20–24*	0.12 (0.10, 0.15)	**<0.001**	0.09 (0.08, 0.12)	**<0.001**
*25–29*	0.31 (0.27, 0.36)	**<0.001**	0.27 (0.23, 0.32)	**<0.001**
*30–34*	0.61 (0.53, 0.69)	**<0.001**	0.54 (0.47, 0.63)	**<0.001**
*35–39*	0.70 (0.61, 0.80)	**<0.001**	0.64 (0.55, 0.74)	**<0.001**
*40–44*	0.87 (0.75, 1.00)	**<0.001**	0.79 (0.68, 0.92)	**0.003**
*45–49 ®*	1.00			
**Respondent education**				
*No formal education*	5.92 (4.80, 7.31)	**<0.001**	2.65 (2.03, 3.45)	**<0.001**
*Primary*	4.28 (3.47, 5.28)	**<0.001**	2.56 (1.98, 3.31)	**<0.001**
*Secondary*	2.36 (1.91, 2.91)	**<0.001**	2.09 (1.64, 2.65)	**<0.001**
*Higher ®*	1.00		1.00	
**Partner education**				
*No formal education*	3.07 (2.69, 3.51)	**<0.001**	1.65 (1.36, 2.00)	**<0.001**
*Primary*	2.45 (2.13, 2.81)	**<0.001**	1.69 (1.41, 2.02)	**<0.001**
*Secondary*	1.67 (1.45, 1.92)	**<0.001**	1.34 (1.14, 1.58)	**<0.001**
*Higher ®*	1.00		1.00	
**Ever experienced child death**				
*No ®*	1.00		1.00	
*Yes*	1.48 (1.35, 1.63)	**<0.001**	0.89 (0.80, 0.99)	**0.026**
**Types of contraception use**				
*No method*	1.14 (1.05, 1.24)	**0.002**	0.93 (0.83, 1.03)	0.171
*Traditional*	1.17 (1.03, 1.33)	**0.017**	0.92 (0.80, 1.06)	0.239
*Modern ®*	1.00		1.00	
**Access to mass media**				
*No*	1.63 (1.51, 1.76)	**<0.001**	1.10 (0.99, 1.22)	0.072
*Yes ®*	1.00		1.00	
***Women empowerment***				
**Number of decision(s) women participate**				
*0*	0.87 (0.73, 1.02)	0.091	0.82 (0.67, 1.01)	0.058
*1–2*	1.08 (0.97, 1.20)	0.170	1.07 (0.94, 1.21)	0.289
*3–4*	1.14 (1.04, 1.25)	**0.004**	1.02 (0.91, 1.14)	0.752
*5 ®*	1.00		1.00	
**Beating Justification**				
*No ®*	1.00		1.00	
*Yes*	1.07 (0.98, 1.16)	0.129	0.92 (0.84, 1.01)	0.087
**Member of any NGO**				
*No*	0.86 (0.80, 0.93)	**<0.001**	0.90 (0.83, 0.99)	**0.026**
*Yes ®*	1.00		1.00	
**Respondent's control over earnings**				
*No earning*	1.02 (0.94, 1.11)	0.652	1.17 (1.07, 1.28)	**0.001**
*No control*	0.91 (0.75, 1.10)	0.319	0.91 (0.74, 1.11)	0.345
*Control ®*	1.00		1.00	

®: Reference category; OR: odds ratio; CI: confidence interval

^1^ Model I represents findings from bivariate logistic regression analysis; and

^2^ Model II shows the findings from the multivariate logistic regression analysis.

In the adjusted model (Model II), it was observed that the highest odds of having more children than desired were in Chittagong division (OR = 2.32; CI: 1.98, 2.73) followed by Sylhet (OR = 2.20; CI: 1.8, 2.7) and Barisal (OR = 1.59; CI: 1.30, 1.95), when compared to Rajshahi division. Significantly, mothers from the poorest quintile were 1.60 times more likely to have more children than they desired (CI: 1.32, 1.92; *P<0*.*001*). Religious belief and mothers’ age were found to be significantly associated with unmet fertility desire, with Hindu mothers having a lower likelihood, and elderly mothers being more likely to have more children than they desired compared to the Muslims and younger mothers. The educational attainment of the respondents and their partners were identified as significant factors for unmet fertility desire. The results revealed that respondents and their partners with no formal education were 2.65 and 1.65 times more likely to have unmet fertility desire than their higher-educated counterparts (CI: 2.03, 3.45; *P < 0*.*001* and CI: 1.36, 2.0; *P < 0*.*001* respectively). Additionally, mothers who had experienced child death were less likely to have unmet fertility desires in the adjusted model; however, the inverse relationship was observed in the unadjusted model (Model I).

Unadjusted model (Model I), based on empowerment-related indicators, our study determined that respondents who participated in three to four household decisions were more likely (OR = 1.14; CI: 1.04, 1.25; *P = 0*.*004*) to have unmet fertility desire while who were not members of any NGOs were less likely to have this (OR = 0.86; CI: 0.80, 0.93; *P = 0*.*001*), however, no significant association was found between beating justification and unmet fertility desire neither of these two models. In the adjusted model (Model II) it was observed that respondents who were not involved in any income-generation activities were significantly more likely (OR = 1.17; CI: 1.07, 1.28; *P = 0*.*001*) to have more children than they desired. Although in the unadjusted model (Model I), women’s participation in household decision making was found significantly associated with the unmet fertility desire, however, no such significant association was found in the adjusted model (Model II).

## Discussion

Bangladesh has made tremendous improvements in reducing TFR substantially to restrict excessive population growth. Despite this, our findings revealed differences between perceived ideal number of the family size and the actual number of children for households. For instance, although the majority of the mothers (71%) perceived two children as an ideal number, only 45% reported that they limit their number of children up to two. About half of the respondents experienced unmet fertility desires having more children than they expected to have initially. Several factors such as geographic location, religion, wealth index, age, education, experiencing child death, and women empowerment indicators were significantly associated with mothers’ desired number of children and unmet fertility desires.

The findings of our study revealed that most mothers (71.2%) expected to have two children for their family, which is in line with the population policy of Bangladesh [25]; however, in reality, approximately 55% of households reported having three or more children in their households at the current stage. Notably, there might be some factors behind having more children than they desired, such as superstitions related to the use of contraceptives, lower level of maternal and partner education, lack of reproductive knowledge, and even a lack of mutual decision-making. One-third of mothers reported not using any contraceptive methods for preventing pregnancy, having no formal education, and having no access to any mass media; moreover, their partners were also not educated or concerned about birth control. Earlier studies indicated that the discontinuation of contraceptive methods may lead to unplanned pregnancies and unwanted births in Bangladesh [26]. A similar study also observed that approximately 33% of pregnancies are unplanned in Bangladesh, which is a crucial factor in the rapid growth of the Bangladeshi population [27]. In resource-poor countries like Bangladesh, children are often considered a precious resource for future economic growth at the household level, as having more children could increase the household's income due to the possibility of engagement of additional family members in the labor market [28,29]. The present study showed that mothers living in Chittagong and Sylhet divisions exhibited a higher tendency to have more children. This finding is supported by earlier studies reporting that geographical variations are important contributors to the unmet need for contraception, and thus fertility [9,30,31]. This may be due to various supply-side factors, such as the distance to health facilities, fragile service delivery, poor communication systems as well as cultural beliefs [9,29,32].

We observed a significant relationship between religion and fertility desire, where women with Muslim religious views have greater unmet fertility needs. This finding is confirmed by previous study findings, where similar associations were also reported. For instance, a study conducted on Saudi women observed that religious prohibition is one of the most significant barriers to using any type of contraceptives [8]. The use of contraceptive methods is a significant factor of unmet need of fertility desire in our study that is related to religious beliefs [26]. Women often choose to prevent pregnancy by using various contraceptive methods for either have a birth interval or to stop childbearing; however, contraceptive discontinuation is very common in developing countries [30,31,33]. Studies observed that the various reasons for limiting contraceptive use include contraception failure, actual or perceived problems with the contraception method, lack of information, religious conservatism, husband’s reluctance, and various supply-side factors (e.g., family planning kit, health education) that strongly contribute to an unplanned pregnancy and unwanted births [8,34]. Therefore, policies should target households with a low uptake of family planning considering both the supply and demand side barriers.

The present study also reveals that there is a significant relationship between the unmet need for fertility and the age and educational status of mothers. The positive relationship between the level of education and public health awareness is well established [9,31,33,35]. Educated people have more knowledge on various health issues including self-care with self-dependency, the benefits of maternal care, and better knowledge regarding the adverse effects of pregnancy-related complications and associated costs; as such, they can lead households in a planned manner [36,37]. Therefore, greater investment is required to reduce inequities in education, particularly for women. Furthermore, knowledge of various health issues is very poor among rural and remote mothers—a trend that was also observed in our study [38]. We found that unmet fertility desire is higher among mothers living in the Sylhet division than their counterparts. Based on evidence from existing literature, we found that most health indicators (e.g., education, contraceptive use, maternal care, and undernutrition) remain poor in the Sylhet region, which is often known as a poor-performing region in the country [35,39,40]. Furthermore, Sylhet is also recognized as a hard-to-reach area since it consists of hilly and flood-prone areas. Communication system is often disrupted for most of the year; therefore, both demand- and supply-side factors might reinforce these findings [39]. The policy should focus on the rural and unprivileged population to reduce the excess burden of more child delivery than desired at the population level. A joint effort involving the government and non-governmental organizations is also necessary to improve the current situation in low-performing areas. This study also observed a positive relationship between older mothers and their unmet fertility needs. Therefore, further qualitative research is recommended to unravel the reasons behind this finding. As per earlier studies, we also observed that the wealth index is a crucial factor for unmet fertility needs, as unmet needs are lower for the wealthiest quintile households [28–31,33,38]. A positive association between health and wealth status has been well established, as mothers from the poorest households have limited access to health facilities and mass media; therefore, they are less likely to be informed of the detrimental effect of excess fertility. On the contrary, higher socioeconomic positions are likely to represent better living conditions with higher affordability, which also contributes to better health [41,42]. Therefore, policymakers should explore and adopt measures to address the aforementioned issues while particularly targeting the poorest and most disadvantaged households for the betterment of maternal health in Bangladesh.

While providing many useful results, the present study had several limitations. First, this study was based on cross-sectional data, which limits the establishment of a causal relationship. Second, this study included only five aspects of women’s participation in decision-making to explore perception, and respondents were only women. As a result, there may be potential discordance regarding the level of autonomy and women’s empowerment status, which may remain underestimated. Therefore, it may be prudent to collect information from both women and men in future studies to generate a more reliable picture of women’s empowerment. However, we were unable to explore men’s perception due to data unavailability in the DHS survey. Despite these limitations, the primary strength of this study includes the national representation of findings, as the latest national representative DHS dataset was used for this analysis. Therefore, the findings remain noteworthy and relevant in drawing attention to policymakers for the betterment of family planning and maternal health.

## Conclusions

Tracking the unmet need for fertility desire is useful for assessing progress towards the target of achieving universal access to reproductive health in Bangladesh. Findings revealed that the perceived ideal number of children differs among women’s socioeconomic and demographic strata. Unmet fertility desire was also found which indicates that reproductive knowledge and health care services are still necessary. Several factors including age, religion, maternal and paternal education, socioeconomic strata, administrative regions and some empowerment-related indicators like participation of household decision making, membership of any NGOs and involvement of any income generation activities were significant influencing factors of unmet fertility desire among Bangladeshi women. Periodical assessment and monitoring the level of unmet fertility desire is recommended particularly in low-performing regions so that all strata of the society can benefit.
